# Microencapsulation of Probiotic Strains by Lyophilization Is Efficient in Maintaining the Viability of Microorganisms and Modulation of Fecal Microbiota in Cats

**DOI:** 10.1155/2020/1293481

**Published:** 2020-07-30

**Authors:** Bruna M. Rodrigues, Paula M. Olivo, Milene P. Osmari, Ricardo S. Vasconcellos, Leonir B. Ribeiro, Ferenc I. Bankuti, Magali S. S. Pozza

**Affiliations:** ^1^Animal Science Department, State University of Maringá, Avenida Colombo, 5790, Maringá, Paraná 87020-900, Brazil; ^2^Federal University of Santa Catarina, Engenheiro Agronômico Andrei Cristian Ferreira St., Florianópolis, Santa Catarina, Brazil

## Abstract

High extrusion temperatures may compromise the functionality of probiotics in dry food. This study aimed to (i) evaluate the effects of two types of microencapsulation techniques, different encapsulating agents, and 120 days of storage on the viability of a commercial probiotic product and (ii) investigate fecal microbiota populations and fecal characteristics of adult cats fed with diets supplemented with probiotics. Three experimental treatments were evaluated: T1, commercial feed (control); T2, commercial kibbles coated with probiotics; and T3, commercial feed supplemented with freeze-dried probiotics and fructooligosaccharides. Fructooligosaccharides and gum arabic were used as encapsulating agents for freeze drying and spray drying and a pool containing *Lactobacillus acidophilus*, *Lactobacillus casei*, *Lactobacillus lactis*, *Bifidobacterium bifidum*, *Enterococcus faecium*, and *Saccharomyces cerevisiae* as a probiotic. Diets were provided to 18 adult cats for 20 days. Feed samples were evaluated microbiologically, and feces were characterized according to their microbial content, pH, and fecal score. Freeze drying was more effective in maintaining microbial viability. Microcapsules prepared using fructooligosaccharides as encapsulants had the highest bacterial count: 8.74 log CFU/g of lactic acid bacteria and 8.75 log CFU/g of enterococci. Probiotics and synbiotics positively modulated (*P* < 0.05) the fecal microbiota of cats, increasing the lactic acid bacteria counts from 3.65 to 4.87 and 5.07 log CFU/g, respectively. Microbial viability decreased significantly (*P* < 0.05) after storage, demonstrating the need for effective protection mechanisms against extrinsic agents. In conclusion, the supplementation of cat diets with probiotics positively affected the gut microbiota. However, the results reinforce that probiotic microorganisms must be incorporated into the animal feed via effective mechanisms to withstand harsh processing conditions and storage.

## 1. Introduction

Cats and dogs are often considered more than household pets; they are esteemed family members. Over the years, not only their population has grown immensely but also the interest in animal health and well-being has grown immensely (Grześkowiak et al.) [1]. Recent efforts have focused on the development of functional foods for pets, such as probiotics, which are live microorganisms that confer health benefits to the host when ingested in adequate quantities (Hill et al.) [2]. The composition of animal gut microbiota may be negatively impacted by antibiotics, gastrointestinal infections, changes in diet, and weaning (Vezquez-Mendonza et al.) [3]. Probiotics have been widely used for the prevention and treatment of disorders and diseases, ranging from allergies to acute gastroenteritis. They have shown a great potential for the control of overweight, urogenital tract infection, and parasitic gastritis, but further research is needed to confirm these applications (Grześkowiak et al.) [1].

The microorganisms most used as probiotics are those belonging to the group of Gram-positive bacteria, that present the outermost portion of the cell wall composed of peptidoglycans and lipoteichoic acid and are more porous (*Lactobacillus*, *Streptococcus*, *Lactococcus*, *Pediococcus*, *Enterococcus*, *Bifidobacterium*, *Bacillus*, and *Clostridium*) (de Melo et al.) [4], Gram negative bacteria, that have in addition to the peptidoglycan, a layer composed of lipopolysaccharides, lipoproteins, and proteins, which act as a physical barrier (nonpathogenic *Escherichia coli*) (Dai et al.) [5], and and yeast (*Saccharomyces*) (Vieira et al.) [6]. In cats, the predominant bacteria come from the phylum Firmicutes, which contains microorganisms such as *Bacillus*, *Enterococcus*, and *Lactobacillus* (Handl et al.) [7], being generally Gram-positive microorganisms.

For functional foods containing probiotics to be eligible to make health claims, microorganisms must be able to resist the processing operations, the handling, the storage, and finally, the passage through the gastrointestinal tract (Souza et al.) [8]. In the pet food industry, processing is a critical determinant of bacterial viability. High temperatures (150–160°C) are required for feed extrusion, which leads to the elimination of microorganisms (Nakandakare et al.) [9].

Few studies have been published on the quality of probiotic products for pets or the ability of microorganisms to survive the digestion process. Dzanis [10] evaluated cat and dog food products containing probiotics and found that microorganisms were absent or present at lower concentrations than those reported by manufacturers. This discrepancy may be due to environmental factors affecting microorganism viability.

To avoid loss of probiotic viability during processing, storage, and digestion, the industry has been seeking alternatives to protect probiotic microorganisms. An interesting strategy is microencapsulation, a process by which probiotics can be covered by microsized encapsulants. Encapsulants are typically insoluble in acidic media but soluble in alkaline media, favoring the release of core contents in the intestine.

In view of the potential benefits of probiotics to intestinal health and the constant search for improved animal health and welfare, we aimed to evaluate the resistance of a commercial probiotic to microencapsulation and its viability during 120 days of storage. A second aim was to evaluate changes in fecal microbiota and fecal characteristics of adult cats fed with diets containing probiotics.

## 2. Materials and Methods

Experimental procedures were approved by the Animal Ethics Committee (CEUA) of the State University of Maringá (under protocol number 3211300517). Two experiments were carried out to evaluate (i) the effects of microencapsulation and storage on probiotic viability and (ii) alteration of fecal microbiota and fecal characteristics of domestic cats fed with diets containing probiotics.

### 2.1. Experiment I: Determination of Probiotic Viability after Microencapsulation and Storage

A commercial probiotic containing 10^9^ colony-forming units (CFU)/g of *Lactobacillus acidophilus*, *Lactobacillus casei*, *Lactobacillus lactis*, *Bifidobacterium bifidum*, *Enterococcus faecium*, and *Saccharomyces cerevisiae* was used. Gum arabic (Fibregum™, CNI Colloides Naturels International, São Paulo, Brazil) and fructooligosaccharides (FOS) (Fosvita, Vitafor, Araçoiaba da Serra, Brazil) were used as encapsulating agents; trehalose (6138-23-4, Sigma-Aldrich, St. Louis, USA) was used as a thermoprotectant for spray drying; and reconstituted skimmed milk (Molico, Nestlé®) diluted in 20% glycerol was used as a cryoprotectant for freeze drying.

Microorganisms were activated prior to microencapsulation by mixing 2% of probiotics in 100 ml of de Man, Rogosa, and Sharpe (MRS) broth (HiMedia™ Laboratories, Mumbai, India) and Brain Heart Infusion (BHI) broth (HiMedia™ Laboratories, Mumbai, India) in an Erlenmeyer flask at 37°C for 24 h. Subsequently, the mixture was incubated with yeast extract peptone dextrose (YEPD) broth (HiEncap™ YEPD, HiMedia Laboratories, Mumbai, India) at 26°C for 24 h in a conventional oven (1399, Pramel®, Campo Bom, Brazil). The contents were transferred to Falcon tubes, centrifuged (Excelsa Baby I-206, Fanem®, São Paulo, Brazil) at 6000 rpm for 10 min at room temperature and then washed twice with sterile water (Liserre, Ré, and Franco) [11]. The bacterial colony was resuspended in 0.2 ml of sterile water.

A solution containing 600 ml of 0.05 M phosphate-buffered saline (PBS, pH 7.6), 1% of activated microorganisms, 2% of trehalose, and 2% of FOS was spray dried (Mini spray dryer B-191, Büchi Labortechnik AG, Flawil, Switzerland) at inlet air temperature of 175°C and at outlet air temperature of 100°C.

Three solutions (S1, S2, and S3) were prepared for freeze drying, differing in the type of encapsulating agent (2% gum arabic, 2% FOS, and 2% gum arabic + 2% FOS, respectively). Solutions were composed of 1% of activated microorganisms, 10% of the cryoprotective solution (20% reconstituted skimmed milk), and 2% of the respective encapsulant in 150 ml of 0.05 M PBS (saline phosphate buffer) (pH 7.6). Solutions were mixed with a magnetic stirrer (78HW-1, Biomixer®, São Paulo, Brazil) for 5 min, then frozen at −20°C, and freeze dried (Liotop L101, Liobras, São Carlos, Brazil) for 48 h.

The commercial probiotic supplement and microcapsules were examined by scanning electron microscopy. Probiotic viability after microencapsulation was determined by plate counting. Microcapsules were added to 0.05 M PBS (pH 7.6) at 0.01 g/ml, then stirred at 150 rpm, 37°C, for 5 min (Grosso and Favaro-Trindade) [12]. Solutions were then diluted in 9 ml of peptone water and seeded in Petri dishes containing MRS agar for *Lactobacillus* and *Bifidobacterium* growth; M17 agar for *Enterococcus* growth (HiMedia Laboratories, Mumbai, India); and YEPD for *Saccharomyces* growth at 37°C for 48–72 h. Colonies were counted using a digital counter (CP600 Plus, Phoenix Luferco®, São Paulo, Brazil), and the results were expressed as log CFU/g.

Microcapsules were subjected to in vitro digestion to evaluate their resistance to passage through the stomach and small intestine. Digestibility coefficients were determined according to the method of Hervera et al. [13]. In short, 1 g of sample was homogenized in 25 ml of 0.1 M PBS (pH 6). The solution received the addition of 10 ml of 0.2 M HCl (hydrochloric acid), and the pH was adjusted to 2 using 1 M HCl and 1 M NaOH. Then, 1 ml of a pepsin solution containing 10 mg of pepsin (3651 U/mg) was added. Samples were incubated in the water bath (MA093, Marconi®, São Paulo, Brazil) at 39°C for 2 h under constant agitation.

After incubation, flasks were cooled and received an addition of 10 ml of 0.2 M PBS (pH 6.8) and 5 ml of 0.6 M NaOH. The pH was adjusted to 6.8 using HCl and NaOH solutions. Then, 1 ml of pancreatin solution containing 100 mg of pancreatin powder was added. Samples were incubated in the water bath, at 39°C, for 4 h under constant agitation. After this period, solutions were seeded in Petri dishes containing MRS, M17, or YEPD and incubated for 48 h, at 35°C, which is the ideal temperature to for the growth of lactic acid bacteria, and 26°C, which is optimal for the yeast.

Three experimental treatments were obtained using a commercial feed (Table 1); T1, commercial feed (control); T2, commercial feed and probiotics; and T3, commercial feed and freeze-dried probiotics with FOS. The experimental diets were coated with 4.5% of poultry oil and 2.0% of liquid palatant (Dtech 12L, SPF Palatability, Descalvado, Brazil). In T2, the probiotics (2 g/cat day) were added by coating, and in T3, the same probiotic dose was added, plus FOS (1 g/cat day), as recommended by the manufacturer for better adhesion. The feed was later provided to the animals for 20 days, and samples of 25 g were used to evaluate the storage time for 120 days.

Sanitized polyethylene packs containing 25 g of feed samples were stored in the dark at room temperature (25°C) for 120 days. For the determination of microbiological parameters, 25 g of feed was diluted in 225 ml of peptone water and then serially diluted in test tubes containing 9 ml of peptone water; finally the feed was seeded in Petri dishes containing MRS, M17, or YEPD agar.

Results of microbial viability and digestibility assays were subjected to analysis of variance (ANOVA), followed by Tukey's test at *P* < 0.05 using SAS version 9 (SAS Institute, North Carolina, USA).

### 2.2. Experiment II: Analysis of Fecal Microbiota and Fecal Characteristics of Adult Cats

Eighteen castrated cats (males, *n* = 9; females, *n* = 9) weighing 3.8 ± 0.56 kg and aged 3 ± 0.84 years were used in this study. The cats were divided between the three experimental treatments: T1, commercial feed (control); T2, commercial feed and probiotics; and T3, commercial feed and freeze-dried probiotics with FOS, with six animals per treatment, equally distributed between treatments in relation to sex and average weight. A randomized block design (2 blocks and 6 animals per treatment) was used. The animal feed was prepared in the first week of the experiment and stored an amount enough for the supply of animals during the period of 20 days. The experiment lasted 20 days, with 15 days of adaptation to the diet and 5 days of feces collection.

The amount of feed supplied was calculated according to the energy requirements of adult cats, as recommended by the National Research Council [14]. During the adaptation period, cats were housed in a 49 m^2^ cattery with *ad libitum* access to water. Cats were housed in individual metal cages (0.5 × 0.5 × 0.6 m) for feeding, which occurred twice a day (8 : 00–10 : 00 a.m. and 2 : 00–4 : 00 p.m.). During the fecal collection period, cats were permanently kept in individual metal cages. Leftover diet was collected and weighed using a digital balance (Prix 3 fit, Toledo do Brazil, São Bernardo do Campo, Brazil).

Regarding the collection period, feces were collected on days 0, 2, and 4. Fecal pH was determined by diluting 4 g of fresh feces in 10 ml of distilled water and then measuring the pH with a digital pH meter (DM20, Digicrom Analytic Ltda, São Paulo, Brazil) (Walter et al.) [15].

Fecal samples were scored according to Carciofi et al. [16], using the following scoring system: 0, liquid feces; 1, pasty and formless feces; 2, soft feces that take on the shape of the container; 3, soft, moist feces that adhere to the container; 4, well-formed, dry feces that do not adhere to the container; and 5, well-formed, hard, dry feces.

For the evaluation of fecal microbiota, 1 ml of feces was diluted in 99 ml of peptone water, then rediluted in 9 ml of peptone water, plated on MRS agar to determine lactic acid bacteria counts and, finally, plated on MacConkey agar (HiMedia Laboratories, Mumbai, India) to determine total coliform counts. Plates were incubated at 35°C for 48 h, and colonies were counted using a digital counter (CP600 Plus, Phoenix Luferco®, São Paulo, Brazil).

Data were subjected to ANOVA, followed by contrast analysis and Tukey's test at *P* < 0.05. Statistical analyses were carried out using SAS version 9 (SAS Institute, North Carolina, USA).

## 3. Results

### 3.1. Experiment I: Effects of Microencapsulation and Storage on Microbial Viability

**Figure 1:**
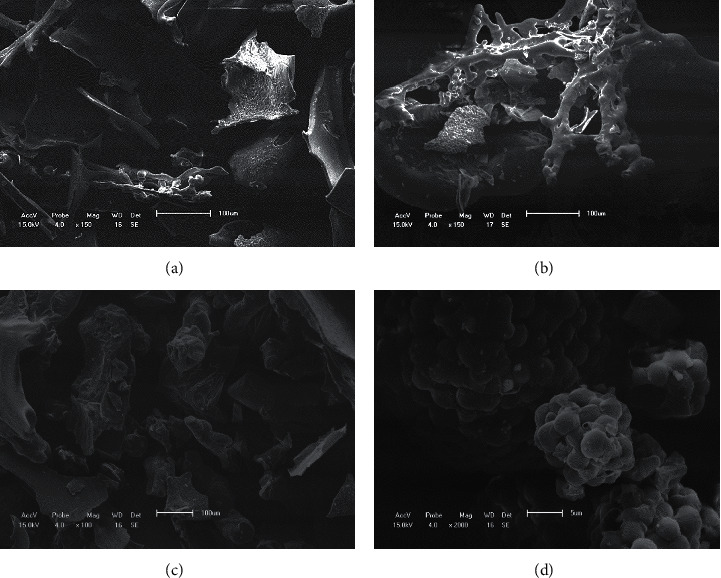
Scanning electron microscopy images of lyophilized microcapsules (A–C) and a commercial probiotic supplement (D). (a) Probiotic microcapsule with fructooligosaccharides as encapsulant (150x magnification); (b) probiotic microcapsule with gum arabic and fructooligosaccharides as encapsulants (100x magnification); (c) probiotic microcapsule with gum arabic as encapsulant (100x magnification); (d) commercial probiotic (2000x magnification).

**Figure 2:**
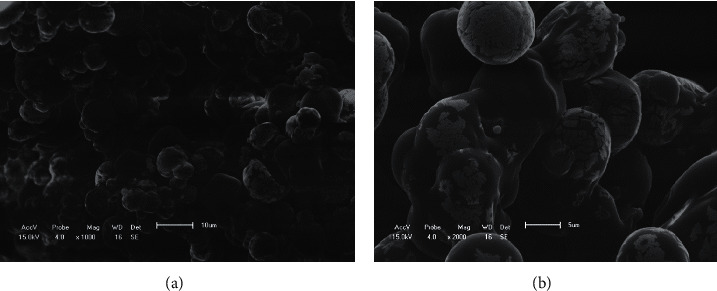
Scanning electron microscopy images of microcapsules prepared by spray drying. (a) 1000x magnification; (b) 2000x magnification.

The commercial probiotic contained 8.25 log CFU/g of lactobacilli, 8.27 log CFU/g of enterococci, and 8.25 log CFU/g of yeasts. Although spray drying produced rounded, uniform, and smooth capsules (Figure 1), microbial viability was not maintained. Freeze drying, in contrast, was effective in maintaining the viability (*P* < 0.0001) but did not produce well-rounded, smooth microcapsules (Figure 2).

The lowest microbial counts were observed in microcapsules prepared using both gum arabic and FOS as encapsulating agents, whereas the highest lactobacilli and enterococci counts were obtained using only FOS as encapsulant (*P* < 0.0001) (Table 2).

Microbial viability reduced sharply after simulated digestion (Table 2). The lowest reduction was observed in microcapsules containing only probiotics: *Lactobacilli* reduced by 11.86%, *Enterococci* by 12.70%, and yeasts by 12.97%. Microcapsules prepared with gum arabic had the highest viability loss (up to 4.32-log reduction) (Table 2).

Overall, microcapsules containing probiotics and FOS showed the best viability results and were therefore added to commercial feed for further assays.

A significant loss in microbial viability in experimental feeds (*P* < 0.0001) was observed after 120 days of storage (Table 3). Viability loss was higher in T2 (commercial feed supplemented with probiotics), with 35.00% reduction in lactobacilli, 30.17% in enterococci, and 36.13% in yeasts. In T3 (commercial feed supplemented with freeze-dried probiotics and FOS), lactobacilli, enterococci, and yeasts were reduced by 21.32%, 21.18%, and 19.02%, respectively (Table 3).

We estimated that the loss in microbial viability of the commercial probiotic supplement after 7 months of storage would be 1.63 log CFU/g for *Lactobacilli*, 1.13 log CFU/g for *Enterococci*, and 1.24 log CFU/g for yeasts (Table 4).

### 3.2. Experiment II: Fecal Microbiota and Fecal Characteristics of Adult Cats

During the second experimental period, the average daily feed consumption was 56, 56, and 54 g/day, for control treatment (T1), probiotic-supplemented feed (T2), and pre- and probiotic-supplemented feed (T3), respectively. These results indicate that, on average, cats consumed the probiotic microorganisms dose of 10^6^ CFU/g of feed, which was within the limits recommended by Roy [17], who describes the concentration of probiotics in products as 10^6^ CFU/g, in order to obtain the desired clinical effects.

There was a significant difference (*P* < 0.0001) in fecal *Lactobacillus* counts and pH between treatments (Table 5). FOS was found to have a positive effect (*P* < 0.05) on the survival of beneficial bacteria. Total coliform counts decreased throughout the stool collection period (days 0, 2, and 4). Contrast analysis revealed that the total coliform counts were significantly lower (*P* < 0.05) on the second day of feces collection (Table 5).

No differences (*P* > 0.05) in fecal score were observed between treatments or collection days; fecal scores were always within the normal range (between 3 and 4) for domestic cats (Carciofi et al.) [13].

## 4. Discussion

### 4.1. Experiment I: Effect of Microencapsulation and Storage on Microbial Viability

The commercial probiotic supplement initially contained high concentrations (10^8^ CFU/g) of lactobacilli, enterococci, and yeasts, but no viable cells were observed after spray drying. These findings are in contrast with the results of Silva et al. [18], who studied the spray-drying microencapsulation of *Bifidobacterium animalis* and *L. acidophilus* and reported that this process did not decrease microbial viability. As noted in our study, Silva et al. [18] also observed that microencapsulation produced highly spherical, well-coated capsules that protected probiotic bacteria from environmental conditions, such as pH, storage, and in vitro simulation of the passage resistance through the gastrointestinal tract. Ananta et al. [19] showed that the higher the drying temperature of *L. rhamnosus* by spray drying, the lower the survival rate. Nunes et al. [20] also observed a reduction in bacterial viability with temperature in the microencapsulation of *B. animalis* Bb-12 and *L. acidophilus* La-5 by spray drying (10°C to 140°C). A reduction of 2.7 log CFU/g was observed immediately after spray drying at 140°C. So, in our study, the high inlet temperature of spray drying (175°C) probably led to microbial unfeasibility after drying.

Spray drying is a rapid and efficient technique capable of producing large amounts of dry material; however, with this technique, the cell viability is reduced (Sunny-Roberts and Knorr) [21]. Protective agents can be used to minimize this problem. Trehalose, a glucose disaccharide, is an effective thermoprotectant at low and high temperatures (Sunny-Roberts and Knorr) [20]. Studying the effects of trehalose on *S. cerevisiae*, Trevisol et al. [22] found that this sugar increases not only the stress resistance of yeast cells but also their fermentation capacity. Su et al. [23] obtained a survival rate of 5% after spray drying *L. rhamnosus* GG at an inlet air temperature of 98°C and at an outlet air temperature of 65°C, using trehalose as thermoprotectant. When calcium was added to the feed solution as additional thermal protection, the survival rate increased to 30%. These data reinforce the importance of choosing an appropriate thermoprotectant to preserve microbial viability during spray drying. In this study, trehalose was not an efficient protective agent, probably because of the high temperatures used (inlet air temperature of 175°C and outlet air temperature of 100°C). Postdrying viability also depends on the intrinsic stress tolerance of probiotic microorganisms, chemical and physical characteristics of encapsulating agents, processing time and pressure, and storage conditions (Chàvez and Ledeboer) [24].

Drying at temperatures close to 0°C is known to enhance microbial stability by reducing the rates of chemical reactions (Nag et al.) [25]. Accordingly, we found that the freeze-drying process preserved the viability of *Lactobacilli* and *Enterococci* (Table 2). The microbial viabilities obtained in this study (about 90%) are higher than those reported by Zayed and Ross [26], when assessing the cryoprotectant properties of milk during the freeze drying process of *Lactobacillus salivarius* (about 22%). These results may be attributed to the cryoprotective effect of FOS and gum arabic. Jantarathin et al. [27] produced microcapsules of *L. acidophilus*, inulin, and sodium alginate by extrusion and obtained 88.19% of cell viability. According to Aslan-Tontul and Erbas [28], the combination of probiotics and prebiotics can improve the resistance of microorganisms to processing conditions. Romano et al. [29] reported that oligosaccharides have a protective effect on probiotics in feed matrices.

Yeast cells are particularly sensitive to freeze drying. Miyamoto-Shinohara et al. [30] investigated the survival rate of different microorganisms during freeze-drying processes and found that *S. cerevisiae* showed a maximum viability of 10%, much lower than that observed for other Gram-negative and Gram-positive bacteria (about 80%).

Freeze-dried probiotics showed considerable loss of viability under simulated gastrointestinal conditions. These data are in agreement with the results of Xu et al. [31] for *L. casei* encapsulated by extrusion followed by freeze drying. The authors observed a relatively low viability reduction (0.41 log CFU/g) after drying, but a significant loss of viability after in vitro gastrointestinal digestion (2.24 log CFU/g). Counterintuitive to these results was the finding of Garcia-Hernandez et al. [32] that probiotic bacteria commonly used in animal feed are greatly resistant to simulated gastrointestinal conditions if they are not previously subjected to other environmental stresses. Thus, the high viability loss observed in this study was probably due to the consecutive stress events: freeze drying followed by hydration, exposure to acidic medium, enzymatic digestion, and alkaline medium.

The probiotic supplement was found to lose viability faster when mixed with feed (Table 5), which might have been caused by the presence of antimicrobial components, pH, and water activity in the feed. Feed mixed with probiotics had a 2-log reduction in *Lactobacillus*, *Enterococcus*, and yeast counts in 120 days of storage, whereas the probiotic supplement took 7 months to achieve a similar loss in viability.

The addition of FOS increased microbial viability in feed after 120 days of storage; that is, the FOS helped to protect probiotic microorganisms from the intrinsic antimicrobial effects of the feed. The protective properties of prebiotics were also observed by Avila-Reyes et al. [33] in the spray-drying microencapsulation of *L. rhamnosus* with inulin and likewise found by Desmond et al. [34] in the encapsulation of *L. paracasei* with gum arabic. González-Forte et al. [35] applied an inulin coating on extruded dog biscuits containing probiotic bacteria (140°C, 45 min) and observed that the protective layer preserved the microbial viability for 30 days of storage.

A combination of different production techniques can be used to protect the microorganisms from environmental conditions and maintain the viability throughout shelf life. Gonçalves et al. [36] added freeze-dried probiotic microcapsules during the preparation of extruded and pelletized dog food and found that bacterial viability remained unchanged for 12 months of storage.

### 4.2. Experiment II: Effect of Probiotics on Fecal Microbiota and Fecal Characteristics of Adult Cats

Probiotics are considered living microorganisms whose ingestion has health benefits; however, there is no minimum concentration established for the inclusion of them in animal feed, so sufficient inclusion is recommended for microorganisms to colonize and bring benefits to the health of the host (Cho and Finocchiaro) [37]. The effectiveness of probiotic products depends on the concentration of viable cells; therefore, there is no lower limit of probiotic counts recommended for animal feed. According to the worldwide recommendation, it is necessary to specify the identification of strains on the label of the feed and the suitable levels of UFC/g. However, the minimum quantities necessary are not defined, but the product is present in an amount which is enough to exercise probiotic function, which must be proven (Brazil) [38]. It was what we noted in this study, in which treatments containing probiotics (T2) or probiotics in combination with FOS (T3) were effective in the increase of beneficial bacteria in the gastrointestinal tract (Table 5). Contrast analysis between T1 (control diet) and T3 confirmed the positive effect (*P* < 0.05) in increasing *Lactobacillus* in the beneficial gastrointestinal microbiota of cats.

There was a reduction in the enteric bacteria during the experimental period, probably because of the increase in the beneficial bacteria, which inhibited the growth of other microorganisms, particularly those not able to ferment FOS, such as *E. coli* and most pathogens (Hidaka et al. [39]; Russell [40]; Swanson et al. [41]; Middelbos et al. [42]). Contrast analysis revealed that total coliform counts were significantly lower on the second day of feces collection. Ritchie et al.) [43], using universal primers, observed in only 13% of fecal samples from cats the presence of *Bifidobacteruim* and *Lactobacillus*, which suggests that these microorganisms are not part of the normal microbiota of these animals, but when they used specific primers to detect *Lactobacillus* and *Bifidobacteria* in fecal samples of cats, they observed the presence of 100% and 92% of the microorganisms in the samples, respectively, so these species are naturally abundant in the cat gastrointestinal tract. Therefore, the dietary supplementation with these microorganisms or with substrates that promote their growth is able to modulate the composition of the gastrointestinal microbiota.

According to Flesch et al. [44], *Lactobacilli* are predominant microorganisms in the small intestine and are responsible for inhibiting the proliferation of pathogenic bacteria by competing for adhesion sites and nutrients and for producing organic acids that reduce intestinal pH. The bifidobacteria are generally prevalent in the large intestine, and they have a beneficial role in preventing diarrhea, in addition to influence the bioavailability and the digestibility of some nutrients in the diet, by lowering the intestinal pH or the presence of iron lactate in the intestine. Another advantageous role is the release of several enzymes in the intestinal lumen, which acts directly on the digestion, thus increasing the absorption of various nutrients, including calcium, magnesium, and iron (Saad, 2006) [45].

According to Silva et al. [46], the probiotic effect of microorganisms is host specific. However, the most commercially available probiotics are not of canine or feline origin. The gastrointestinal tract of these animals certainly rich in microorganisms with a probiotic potential is waiting to be discovered.

The positive effects of probiotics on animal intestinal health were confirmed by Marshall-Jones et al. [47]. The authors supplemented healthy adult cats with *L. acidophilus* DSM13241 and observed an increase in *Lactobacillus* counts in feces, accompanied by a decrease in *Clostridium* spp. and *Enterococcus faecalis*, pH reduction, and beneficial systemic and immunomodulatory effects in cats. In a study with dogs and cats, Bybee et al. [48] found a lower incidence of diarrhea in animals fed with diets enriched with *Enterococcus faecium* SF68, suggesting a positive effect of the probiotic on the gastrointestinal tract of animals. A similar beneficial effect was reported by Garcia-Mazcorro et al. [49], who administered a combination of seven probiotics bacteria (*Enterococcus faecium*, *Streptococcus thermophilus*, *Bifidobacterium longum*, *Lactobacillus acidophilus*, *Lactobacillus rhamnosus*, *Lactobacillus plantarum*, and *Lactobacillus bulgaricus*) and FOS to dogs and cats. High counts of probiotic bacteria, mainly *Enterococcus* e *Streptococcus*, were found in feces, and no adverse gastrointestinal effects were observed.

Gómez-Gallego et al. [50] added fermented milk containing *Lactobacillus fermentum*, *L. rhamnosus*, and *L. plantarum* to the diet of dogs suffering from acute diarrhea. The results were increased appetite, reduced vomiting, and reduced diarrhea, which are effects attributed to the 1.89 log CFU/g reduction in *Clostridium perfringens* in feces samples compared with those of the control (placebo).

Prebiotics can also modify the microbiota of dogs and cats, as made evident by the increase in lactic acid bacteria in feces samples of cats fed with diets containing probiotics and FOS. These results agree with those found by Kanakupt et al. [51], who observed an increase in *Bifidobacterium* spp. in the feces of cats supplemented with 0.5% FOS.

The fecal pH was expected to decrease with the increase in lactic acid bacteria, although in this study, there was no difference regarding the pH values observed in the control treatment and in the treatment supplemented with probiotic and FOS. High concentrations of probiotic bacteria may increase the levels of organic acids, which are resultant from fermentation. Organic acids reduce the pH of the medium, and, together with prebiotics and other antibacterial substances, such as bacteriocins, hydrogen peroxide, and enzymes, they inhibit the growth of pathogenic microorganisms (Tripathi and Giri) [52].

The fecal score is a good indicator of fecal quality, as it indicates the consistency, shape, and moisture of feces. Probiotics and synbiotics did not alter fecal score, which was maintained between 3 and 4, being considered normal. Similar results were reported by Swanson et al. [41], who supplemented dog diets with mannooligosaccharide (MOS) and FOS, and any differences in fecal quality were not observed.

The effectiveness of probiotic products depends on the concentration of viable cells and several factors influence probiotic viability in feed during processing and storage, including feed composition, microbiological parameters, and processing conditions (Thipathi and Giri) [52]. In this study, probiotic and synbiotic supplements were added to the feed only after processing, which suggests that intrinsic feed parameters, such as pH, acidity, redox potential, and water activity, and the concentration of salt, sugar, preservatives, and artificial colorings are responsible for the considerable reduction in probiotic viability during storage.

## 5. Conclusions

The combination of probiotics and FOS improved microbial viability after freeze drying. Supplementation of cat diets with probiotic bacteria was efficient in modulating the gut microbiota. However, probiotic viability in feed decreased during the storage because of the microbial susceptibility to environmental conditions and the incompatibility between microorganisms and feed components, revealing the need for protective formulations and mild production conditions.

## Figures and Tables

**Table 1:** Diet composition.

Ingredient	% of feed
Corn grain	30.40
Poultry meal	29.00
Soybean meal 45%	11.00
Brewers' rice	7.00
Fish meal 61%	5.00
Wheat bran	7.00
Poultry oil	4.50
Liquid palatant	2.00
Beet pulp	1.00
Yeast extract	0.80
Sodium chloride	0.56
Flaxseed	0.40
Vitamin-mineral premix^1^	0.40
Calcium propionate^2^	0.15
Sodium hexametaphosphate^3^	0.10
dl-Methionine	0.10
Odor adsorbent (*Yucca schidigera* extract)	0.08
Mycotoxin adsorbent	0.05
Chelated zinc	0.04
Urinary acidifier^4^	0.40
Synthetic antioxidant^5^	0.02

Metabolizable energy	3500 kcal·kg^−1^

Guaranteed levels: moisture, 12% (max.); crude protein, 30% (min.); ethereal extract, 10% (min.); fibrous matter, 3.5% (max.); mineral matter, 8.5% (max.); calcium, 0.9% (min.); calcium, 1.8% (max.); phosphorus: 0.9% (min.); linolenic acid: 2,500 mg/kg (min.); linoleic acid 25 g/kg (max.); saponin, 4 mg/kg (min.). ^1^The following was provided per kilogram in the diet: vitamin A, 10,000 IU; vitamin D, 720 IU; vitamin E, 48 IU; vitamin K, 0.80 mg; vitamin B_1_, 5.6 mg; vitamin B_2_, 6 mg; vitamin B_6_, 4.8 mg; vitamin B_12_, 22 mg; pantothenic acid, 6 mg; folic acid, 0.80 mg; copper, 5 mg; iron, 80 mg; manganese, 8 mg; iodine, 0.60 mg; zinc, 80 mg; selenium, 0.10 mg; biotin, 0.08 mg; niacin, 64 mg; taurine, 1,000 mg; choline, 2,400 mg. ^2^Antifungal agent. ^3^For tartar control. ^4^Propionic acid. ^5^Butylated hydroxyanisole (BHA) and butylated hydroxytoluene (BHT).

**Table 2:** Microbial count (log CFU/g) of a commercial probiotic product and lyophilized probiotics before and after in vitro digestion.

Microorganism	Before in vitro digestion						After in vitro digestion					
	Prob	S1	S2	S3	SEM	*P*	Prob	S1	S2	S3	SEM	*P*
Lactobacilli	8.25^b^	7.71^c^	7.52^d^	8.74^a^	0.039	<0.0001	7.28^a^	3.67^b^	2.24^c^	3.67^b^	0.039	<0.0001
Enterococci	8.27^b^	8.10^b^	7.86^c^	8.75^a^	0.051	<0.0001	7.22^a^	3.78^c^	3.73^c^	4.65^b^	0.051	<0.0001
Yeasts	8.25^a^	<1.00^b^	<1.00^b^	<1.00^b^	0.034	<0.0001	7.18^a^	<1.00^b^	<1.00^b^	<1.00^b^	0.034	<0.0001

Means in a row followed by different lowercase letters differ by Tukey's test (*P* < 0.05). Prob: commercial probiotic product; S1: solutions containing 1% of activated microorganisms + 10% of the cryoprotective solution + 2% of the gum arabic, S2: solutions containing 1% of activated microorganisms + 10% of the cryoprotective solution + 2% FOS + 2% gum Arabic, and S3: solutions containing 1% of activated microorganisms + 10% of the cryoprotective solution + 2% FOS; SEM: standard error of the mean.

**Table 3:** Microbial viability (log CFU/g) in feed during storage at room temperature.

Storage (day)	Treatment		SEM	*P* value		
	T1	T2		Treatment	Storage	Treatment × storage
*Lactobacilli*						
0	6.52^a^	6.05^b^	0.027	<0.0001	<0.0001^1^	<0.0001
7	5.99^b^	5.28^c^	0.027			
15	5.33^c^	5.26^c^	0.027			
30	5.13^d^	4.94^e^	0.027			
60	4.94^e^	4.97^e^	0.027			
90	4.44^h^	4.86^f^	0.027			
120	4.24^i^	4.76^g^	0.027			

*Enterococci*						
0	6.63^a^	6.28^b^	0.041	<0.0001	<0.0001^2^	<0.0001
7	6.32^b^	5.82^d^	0.041			
15	5.92^cd^	5.67^e^	0.041			
30	5.85^d^	5.97^c^	0.041			
60	5.36^f^	5.60^e^	0.041			
90	5.15^g^	5.02^h^	0.041			
120	4.63^i^	4.95^h^	0.041			

*Yeasts*						
0	6.56^a^	6.10^b^	0.037	0.3030	<0.0001^3^	<0.0001
7	6.17^b^	5.66^d^	0.037			
15	5.77^c^	5.43^e^	0.037			
30	5.15^f^	5.13^f^	0.037			
60	5.01^g^	5.01^g^	0.037			
90	4.69^h^	5.12^f^	0.037			
120	4.19^i^	4.94^g^	0.037			

Means in a column followed by different lowercase letters differ by Tukey's test (*P* < 0.05). T1: feed supplemented with a commercial probiotic product; T2: feed supplemented with freeze-dried probiotics and fructooligosaccharides; SEM: standard error of the mean. ${}^{1}\hat{y}$ = 5.735 − 0.012*x* (*r*^2^ = 66.99%). ${}^{2}\hat{y}$ = 6.218 − 0.012*x* (*r*^2^ = 86.08%). ${}^{3}\hat{y}$ = 5.922 − 0.012*x* (*r*^2^ = 71.17%).

**Table 4:** Microbial viability (log CFU/g) of a commercial probiotic supplement after 7 months of storage.

Microorganism	May 2017	December 2017	% Reduction
*Lactobacilli*	8.28	6.65	19.66
*Enterococci*	8.22	7.09	13.75
Yeasts	8.22	6.98	15.09

**Table 5:** Microbial composition (log CFU/g), fecal pH, and fecal score of cats fed with diets supplemented with probiotics.

Variable	Treatment			SEM	*P*	Contrasts		Time (day)			SEM	*P*	Contrasts	
	1	2	3			T1 × T3	T2 × T1, T3	0	2	4			D0 × D4	D2 × D0, D4
*Lactobacilli*	3.65^b^	5.07^a^	4.87^a^	0.097	<0.0001	<0.0001	<0.0001	4.42	4.61	4.54	0.097	0.3650	NS	NS
Coliforms	2.50	3.00	2.98	0.195	0.1268	NS	NS	3.25^a^	2.31^b^	2.91^ab^	0.195	<0.05	NS	<0.05
Fecal pH	5.67^b^	5.96^a^	5.81^ab^	0.059	<0.05	<0.05	<0.05	5.78	5.88	5.82	0.059	0.4674	NS	NS
Fecal score	3.22	3.22	3.11	0.103	0.6815	NS	NS	3.11	3.19	3.25	0.103	0.6344	NS	NS

Means in a row followed by different lowercase letters differ by Tukey's test (*P* < 0.05). SEM: standard error of the mean; NS: not significant; T1: commercial feed (control); T2: commercial feed and probiotics; T3: commercial feed and freeze-dried probiotics with fructooligosaccharides; D0: day 0; D2: day 2; D4: day 4.

## Data Availability

The data used to support the findings of this study are included within the article.
